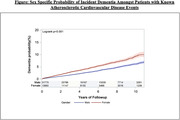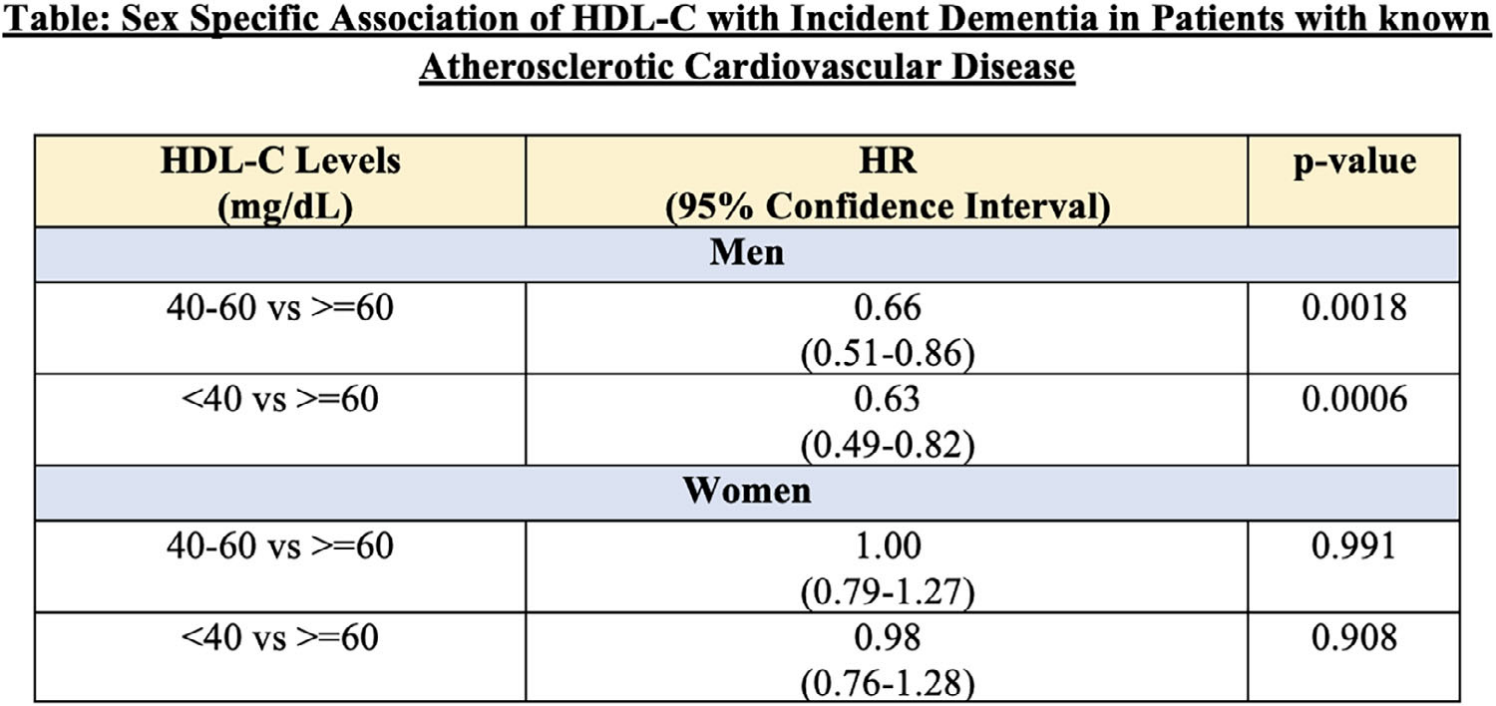# Association of High Density Lipoprotein Cholesterol with Incident Dementia in Patients with Cardiovascular Disease: Insights from a Heart‐Brain Registry

**DOI:** 10.1002/alz.093193

**Published:** 2025-01-09

**Authors:** Anum Saeed, Jianhui Zhu, Floyd Thoma, Oscar L. Lopez, Tharick Ali Pascoal, Suresh Mulukutla, Ann D Cohen, Steven E. Reis

**Affiliations:** ^1^ University of Pittsburgh, Pittsburgh, PA USA; ^2^ UPMC, Pittsburgh, PA USA; ^3^ University of Pittsburgh Alzheimer’s Disease Research Center (ADRC), Pittsburgh, PA USA

## Abstract

**Background:**

Low levels of high density lipoprotein cholesterol (HDL‐c) are a risk factors for atherosclerotic cardiovascular disease (ASCVD). ASCVD can increase the risk for dementia. However, the link between HDL‐C and incidence of dementia remain less clear specifically in women. We investigated the association of HDL‐C and dementia incidence in a contemporary healthcare network electronic health record (EHR) based registry of patients with known ASCVD, stratified by sex.

**Methods:**

Data were abstracted from EHR (2010‐20) in patients ≥50y) with known ASCVD (coronary revascularization or coronary artery bypass grafting) and followed for dementia incidence (based on ICD9/10 codes). Baseline HDL‐C levels risk (for dementia incidence was assessed using Cox proportional hazard ratios across follow up period, adjusted for traditional ASCVD risk factors and statin use. Chi‐square test was used for sex‐stratified analysis.

**Results:**

Among 47,210 patients (70% men vs 30% women; p <0.001) with established ASCVD, 1,725 (3.7%) new dementia cases were documented over a median follow up of ∼5 yr. Women had a higher risk of dementia incidence (HR 1.26, 95% CI [1.13‐1.39] p<0.001) **[Figure]** and a higher mean HDL‐C (47.7 ± 13.4 vs 41.8 ± 12.5 mg/dL, p<0.001) compared to men.

In categorical analysis, lower HDL‐C was associated with an elevated risk of dementia incidence [HR_HDL‐c <40vs ≥60_: 0.81; (95% CI: 0.67‐0.98); p‐value 0.03]. However, when stratified by sex, lower HDL‐C was associated with increased risk of dementia incidence for men (p for interaction <0.05) **[Table]**.

**Conclusion:**

The relationship of high density lipoprotein cholesterol levels with dementia incidence was sex dependent in this contemporary patient cohort of known ASCVD. More research is needed to evaluate the lipoprotein profiles of patients with ASCVD who are at higher risk for dementia.